# Therapeutics Role of* Azadirachta indica* (Neem) and Their Active Constituents in Diseases Prevention and Treatment

**DOI:** 10.1155/2016/7382506

**Published:** 2016-03-01

**Authors:** Mohammad A. Alzohairy

**Affiliations:** Department of Medical Laboratories, College of Applied Medical Sciences, Qassim University, P.O. Box 6699, Buraidah, Saudi Arabia

## Abstract

Neem (*Azadirachta indica*) is a member of the Meliaceae family and its role as health-promoting effect is attributed because it is rich source of antioxidant. It has been widely used in Chinese, Ayurvedic, and Unani medicines worldwide especially in Indian Subcontinent in the treatment and prevention of various diseases. Earlier finding confirmed that neem and its constituents play role in the scavenging of free radical generation and prevention of disease pathogenesis. The studies based on animal model established that neem and its chief constituents play pivotal role in anticancer management through the modulation of various molecular pathways including p53, pTEN, NF-*κ*B, PI3K/Akt, Bcl-2, and VEGF. It is considered as safe medicinal plants and modulates the numerous biological processes without any adverse effect. In this review, I summarize the role of* Azadirachta indica* in the prevention and treatment of diseases via the regulation of various biological and physiological pathways.

## 1. Introduction

The plant product or natural products show an important role in diseases prevention and treatment through the enhancement of antioxidant activity, inhibition of bacterial growth, and modulation of genetic pathways. The therapeutics role of number of plants in diseases management is still being enthusiastically researched due to their less side effect and affordable properties. It has been accepted that drugs based on allopathy are expensive and also exhibit toxic effect on normal tissues and on various biological activities. It is a largely accepted fact that numerous pharmacologically active drugs are derived from natural resources including medicinal plants [1, 2]. Various religious documents such as Bible and Quran also supported the herbs role in health care and prevention. Islamic perspective also confirms the herbs role in diseases management and Prophet Mohammed (PBUH) recommended various plants/fruits in the diseases cure [3]. Neem ingredients are applied in Ayurveda, Unani, Homeopathy, and modern medicine for the treatment of many infectious, metabolic, or cancer diseases [4, 5]. Different types of preparation based on plants or their constituents are very popular in many countries in diseases management. In this vista, neem (*Azadirachta indica*), a member of the Meliaceae family, commonly found in India, Pakistan, Bangladesh, and Nepal, has therapeutics implication in diseases cure and formulation based on the fact that neem is also used to treat various diseases.* Azadirachta indica* has complex of various constituents including nimbin, nimbidin, nimbolide, and limonoids and such types of ingredients play role in diseases management through modulation of various genetic pathways and other activities. Quercetin and ß-sitosterol were first polyphenolic flavonoids purified from fresh leaves of neem and were known to have antifungal and antibacterial activities [6]. Numerous biological and pharmacological activities have been reported including antibacterial [7], antifungal [8], and anti-inflammatory. Earlier investigators have confirmed their role as anti-inflammatory, antiarthritic, antipyretic, hypoglycemic, antigastric ulcer, antifungal, antibacterial, and antitumour activities [9–12] and a review summarized the various therapeutics role of neem [13]. This review summarizes the role of neem and its active ingredients in the diseases prevention and treatment through the modulation of various biological pathways.

## 2. Botanical Description of Neem

Neem tree belongs to the family Meliaceae which is found in abundance in tropical and semitropical regions like India, Bangladesh, Pakistan, and Nepal. It is a fast-growing tree with 20–23 m tall and trunk is straight and has a diameter around 4-5 ft. The leaves are compound, imparipinnate, with each comprising 5–15 leaflets. Its fruits are green drupes which turn golden yellow on ripening in the months of June–August. Taxonomic position of* Azadirachta indica* (neem) is classified in Table 1 [14].

## 3. Active Compounds of *Azadirachta indica* L. (Neem)

*Azadirachta indica* L. (neem) shows therapeutics role in health management due to rich source of various types of ingredients. The most important active constituent is azadirachtin and the others are nimbolinin, nimbin, nimbidin, nimbidol, sodium nimbinate, gedunin, salannin, and quercetin. Leaves contain ingredients such as nimbin, nimbanene, 6-desacetylnimbinene, nimbandiol, nimbolide, ascorbic acid, n-hexacosanol and amino acid, 7-desacetyl-7-benzoylazadiradione, 7-desacetyl-7-benzoylgedunin, 17-hydroxyazadiradione, and nimbiol [15–17]. Quercetin and ß-sitosterol, polyphenolic flavonoids, were purified from neem fresh leaves and were known to have antibacterial and antifungal properties [6] and seeds hold valuable constituents including gedunin and azadirachtin.

## 4. Mechanism of Action of Active Compounds

Neem (*Azadirachta indica*), a member of the Meliaceae family, has therapeutics implication in the diseases prevention and treatment. But the exact molecular mechanism in the prevention of pathogenesis is not understood entirely. It is considered that* Azadirachta indica* shows therapeutic role due to the rich source of antioxidant and other valuable active compounds such as azadirachtin, nimbolinin, nimbin, nimbidin, nimbidol, salannin, and quercetin.

Possible mechanism of action of* Azadirachta indica* is presented as follows.

Neem (*Azadirachta indica*) plants parts shows antimicrobial role through inhibitory effect on microbial growth/potentiality of cell wall breakdown. Azadirachtin, a complex tetranortriterpenoid limonoid present in seeds, is the key constituent responsible for both antifeedant and toxic effects in insects [18]. Results suggest that the ethanol extract of neem leaves showed* in vitro* antibacterial activity against both* Staphylococcus aureus* and MRSA with greatest zones of inhibition noted at 100% concentration [19].

1. Neem plays role as free radical scavenging properties due to rich source of antioxidant. Azadirachtin and nimbolide showed concentration-dependent antiradical scavenging activity and reductive potential in the following order: nimbolide > azadirachtin > ascorbate [20].
2. Neem ingredient shows effective role in the management of cancer through the regulation of cell signaling pathways. Neem modulates the activity of various tumour suppressor genes (e.g., p53, pTEN), angiogenesis (VEGF), transcription factors (e.g., NF-*κ*B), and apoptosis (e.g., bcl2, bax).
3. Neem also plays role as anti-inflammatory via regulation of proinflammatory enzyme activities including cyclooxygenase (COX), and lipoxygenase (LOX) enzyme.

## 5. Therapeutic Implications of Neem and Its Various Ingredients in Health Management

**Figure 1:**
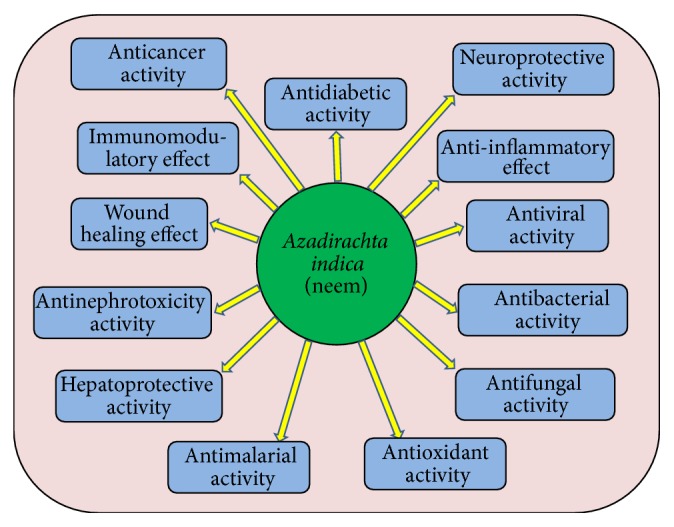
Pharmacological activities of* Azadirachta indica* L. neem in diseases management through the modulation of various activities.

Active constitutes play role in the diseases cure via activation of antioxidative enzyme, rupture the cell wall of bacteria and play role as chemopreventive through the regulation of cellular pathways. Pharmacological activities of neem are discussed in detail (Figure 1).

### 5.1. Antioxidant Activity

Free radical or reactive oxygen species are one of the main culprits in the genesis of various diseases. However, neutralization of free radical activity is one of the important steps in the diseases prevention. Antioxidants stabilize/deactivate free radicals, often before they attack targets in biological cells [21] and also play role in the activation of antioxidative enzyme that plays role in the control of damage caused by free radicals/reactive oxygen species. Medicinal plants have been reported to have antioxidant activity [22]. Plants fruits, seeds, oil, leaves, bark, and roots show an important role in diseases prevention due to the rich source of antioxidant.

Leaf and bark extracts of* A. indica* have been studied for their antioxidant activity and results of the study clearly indicated that all the tested leaf and bark extracts/fractions of neem grown in the foothills have significant antioxidant properties [23]. Another important study was performed based on leaves, fruits, flowers, and stem bark extracts from the Siamese neem tree to assess the antioxidant activity and results suggest that extracts from leaf, flower, and stem bark have strong antioxidant potential [24].

A valuable study was carried out to evaluate* in vitro* antioxidant activity in different crude extracts of the leaves of* Azadirachta indica* (neem) and antioxidant capacity of different crude extracts was as follows: chloroform > butanol > ethyl acetate extract > hexane extract > methanol extract. Result of the current finding suggested that the chloroform crude extracts of neem could be used as a natural antioxidant [20].

Other results revealed that azadirachtin and nimbolide showed concentration-dependent antiradical scavenging activity and reductive potential in the following order: nimbolide > azadirachtin > ascorbate. Furthermore, administration of azadirachtin and nimbolide inhibited the development of DMBA-induced HBP carcinomas through prevention of procarcinogen activation and oxidative DNA damage and upregulation of antioxidant and carcinogen detoxification enzymes [25]. Experimentation was made to evaluate the antioxidant activity of the flowers and seed oil of neem plant* Azadirachta indica* A. Juss. and results revealed that ethanolic extract of flowers and seed oil at 200 *μ*g/mL produced the highest free radical scavenging activity with 64.17 ± 0.02% and 66.34 ± 0.06%, respectively [26].

The results of the study revealed that root bark extract exhibited higher free radical scavenging effect with 50% scavenging activity at 27.3 *μ*g/mL and total antioxidant activity of this extract was found to be 0.58 mM of standard ascorbic acid [27]. Other results of study concluded that tested leaf and bark extracts/fractions of neem grown in the foothills (subtropical region) have significant antioxidant properties [23].

Leaves, fruits, flowers, and stem bark extracts from the Siamese neem tree were evaluated for antioxidant and results of the study showed that leaf aqueous extract and flower and stem bark ethanol extracts showed higher free radical scavenging effect with 50% scavenging activity at 26.5, 27.9, and 30.6 microg/mL, respectively. Furthermore, total antioxidant activity of extracts was found to be 0.959, 0.988, and 1.064 mM of standard trolox, respectively [28].

### 5.2. Anticancerous Activity

**Figure 2:**
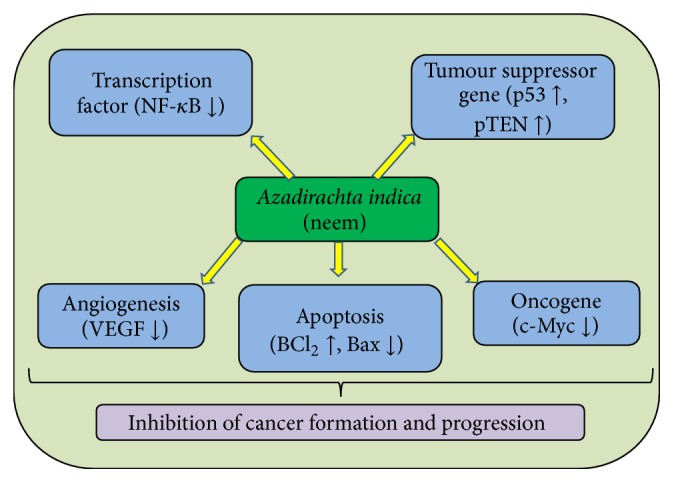
Anticancerous activities of* Azadirachta indica* L. neem through the modulation of various cell signaling pathways.

Cancer is multifactorial disease and major health problem worldwide. The alteration of molecular/genetic pathways plays role in the development and progression of cancer. The treatment module based on allopathic is effective on one side but also shows adverse effect on the normal cell. Earlier studies reported that plants and their constituents show inhibitory effects on the growth of malignant cells via modulation of cellular proliferation, apoptosis, tumour suppressor gene, and various other molecular pathways [29]. Neem contains flavanoids and various other ingredients that play an important role in inhibition of cancer development (Figure 2). Large number of epidemiological studies proposes that high flavonoid intake may be correlated with a decreased risk of cancer [30].

Neem oil holds various neem limonoids which prevents mutagenic effects of 7,12-dimethylbenz(a)anthracene [31]. A study was performed to investigate the cytotoxic effects of nimbolide found in leaves and flowers on human choriocarcinoma (BeWo) cells and results showed that treatment with nimbolide resulted in dose- and time-dependent inhibition of growth of BeWo cells with IC_50_ values of 2.01 and 1.19 *μ*M for 7 and 24 h, respectively [32]. A study was made to assess the chemopreventive potential of the limonoids, azadirachtin, and nimbolide and results showed that azadirachtin and nimbolide inhibited the development of DMBA-induced HBP carcinomas through influencing multiple mechanisms such as prevention of procarcinogen activation and oxidative DNA damage, upregulation of antioxidant and carcinogen detoxification enzymes, and inhibition of tumour invasion and angiogenesis [25].

*Azadirachta indica* and their active compounds play pivotal role in the prevention of cancer development and progression. The exact molecular mechanism in this vista is not understood fully. Based on experimentation, it was considered that neem and its ingredients play role in the modulation of various cell signaling pathways.* Azadirachta indica* hold various ingredients and theses constituents activate the tumour suppressor genes and inactivate the activity of several genes involved in the cancer development and progression such as VEGF, NF-*κ*B, and PI3K/Akt. Neem has been reported to be a good activator of tumour suppressor gene and inhibitor of VEGF and phosphoinositol PI3K/Akt pathways. It also activates apoptosis, suppression of NF-*κ*B signaling, and cyclooxygenase pathway.

Neem and its constituents play role in the prevention of malignancies through the modulation of molecular pathways which are described below.

#### 5.2.1. Effect of Neem and Its Constituents on Tumour Suppressor Genes

p53 is an important tumour suppressor gene and it plays role in the inhibition of the proliferation of abnormal cells, in that way inhibiting the development and progression of cancer. A study confirmed that ethanolic fraction of neem leaf (EFNL) treatment effectively upregulated the proapoptotic genes and proteins including p53, Bcl-2-associated X protein (Bax), Bcl-2-associated death promoter protein (Bad) caspases, phosphatase and tensin homolog gene (pTEN), and c-Jun N-terminal kinase (JNK) [33]. A finding showed that ethanolic neem leaf extract enhanced the expression of proapoptotic genes, such as caspase-8 and caspase-3, and suppressed the expression of Bcl-2 and mutant p53 in the 7,12-dimethylbenz(a)anthracene-induced cancer cells [34, 35].

Nimbolide, a tetranortriterpenoid limonoid, is one of the important contributors to the cytotoxicity of neem extracts [36]. Nimbolide downregulated cell survival proteins, including I-FLICE, cIAP-1, cIAP-2, Bcl-2, Bcl-xL, survivin, and X-linked inhibitor of apoptosis protein, and upregulated the proapoptotic proteins p53 and Bax [37].

pTEN activity is commonly lost via mutations, deletions, or promoter methylation silencing in various types of primary and metastatic cancers [38, 39]. Inactivation of pTEN has been noticed in various types of tumour. A study confirmed that ethanolic fraction of neem leaf treatment significantly increased the expression of pTEN, which could inhibit mammary tumourigenesis through its inhibitory effect on Akt [33].

#### 5.2.2. Effect of Neem and Its Constituents on Apoptosis

bcl2 and bax play an important role in the regulation of apoptotic process. Any alteration in bcl2 and bax causes the development and progression of tumours [40]. Altered expression of such genes has been noticed in many tumours. A study was performed to investigate the effect of extract in an* in vivo* 4T1 breast cancer model in mice and results confirmed that CN 250 and CN 500 groups had a higher incidence of apoptosis compared with the cancer controls [41]. Another study reported that extract has been shown to cause cell death of prostate cancer cells (PC-3) via inducing apoptosis [42].

A study finding revealed that leaf extract downregulated Bcl-2 expression and upregulated Bim, caspase-8, and caspase-3 expression in the buccal pouch indicating that it has apoptosis inducing effects in the target organ [35] and study results confirmed that leaf extract induced a dose-dependent reduction in chronic lymphocytic leukemia (CLL) cell viability with significant apoptosis observed at 0.06% (w/v) by 24 h [43]. Isolated compound and chief constituents from neem show a range of activities affecting multiple targets and also play role in the induction of apoptotic cell death in cancer [44, 45].

#### 5.2.3. Effect of Neem and Its Constituents on Angiogenesis

Angiogenesis is complex process that supplies blood to the tissue and that is essential for growth and metastasis of tumour. Angiogenesis is regulated by activators as well as inhibitors. The development of antiangiogenic agents to block new blood vessel growth is crucial step in the inhibition/prevention of tumour growth. Medicinal plants and their ingredients play role in prevention of tumour growth due to their antiangiogenic activity.

An important study revealed that ethanolic fraction of neem leaf (EFNL) treatment effectively inhibited the expression of proangiogenic genes, vascular endothelial growth factor A, and angiopoietin, indicating the antiangiogenic potential of EFNL. Furthermore, inhibition of angiogenesis by ethanolic fraction of neem leaf (EFNL) could be a reason for reduction in mammary tumour volume and for blocked development of new tumours as observed in current studies [33]. Another study was performed to evaluate the antiangiogenic activity of extract of leaves in human umbilical vein endothelial cells (HUVECs) and results showed treatment of HUVECs with EENL inhibited VEGF induced angiogenic response* in vitro* and* in vivo* and also EENL suppressed the* in vitro* proliferation, invasion, and migration of HUVECs [46]. A study was made on zebra fish embryos via treatment of various concentrations of water soluble fractions of crude methanolic extract of neem root, imatinib (standard), and control and results of the study concluded that water soluble fractions of methanolic extract of neem root were found to have the ability to inhibit angiogenesis [47].

#### 5.2.4. Effect of Neem on Oncogene

An oncogene is a mutated gene that plays significant role in the development and progression of tumours. Experiment was performed to investigate effect of leaf extract on c-Myc oncogene expression in 4T1 breast cancer BALB/c mice and results revealed that 500 mg/kg neem leaf extract (C500) group showed significant suppression of c-Myc oncogene expression as compared to the cancer control group [48].

#### 5.2.5. Effect of Neem on PI3K/Akt Pathways

PI3K/Akt pathways show pivotal effect in the promotion of tumour. However, inhibition of PI3K/Akt pathways is one of the important steps towards regulation of tumour development. Effect of leaf extract on PI3K/Akt and apoptotic pathway in prostate cancer cell lines (PC-3 and LNCaP) was investigated and results suggested that effect of leaf extract induces apoptosis and inhibits cell proliferation through inhibiting PI3K/Akt pathway in both PC-3 and LNCaP cells [49].

Another study was performed to evaluate the molecular mechanisms involved in the induction of apoptosis and antiproliferative activity exerted by leaf extract on the human breast cancer cell lines and results confirmed that extract treated cells significantly decreased the protein expression such as IGF signaling molecules IGF-1R, Ras, Raf, p-Erk, p-Akt, and cyclin D1  [50].

Another study was carried out to evaluate the effects of nimbolide on apoptosis and insulin-like growth factor (IGF) signaling molecules in androgen-independent prostate cancer (PC-3) cells line and results of the study suggested that nimbolide acts as a potent anticancer agent by inducing apoptosis and inhibiting cell proliferation via PI3K/Akt pathway in PC-3 cells [51].

#### 5.2.6. Effect of Neem on NF-*κ*B Factor

The NF-*κ*B transcription factor plays a major role in cancer and related diseases [52]. However, the inhibition of NF-*κ*B action is a vital step in the prevention of cancer development and progression. An important study was performed to investigate the efficacy of bioactive phytochemicals in inhibiting radiotherapy- (RT-) induced NF-*κ*B activity, signaling, and NF-*κ*B-dependent regulation of cell death and results showed that curcumin, leaf extract, and black raspberry extract (RSE) significantly inhibited both constitutive and RT-induced NF-*κ*B [53] and other important study results demonstrate that nimbolide, a neem derived tetranortriterpenoid, concurrently abrogates canonical NF-*κ*B and Wnt signaling and induces intrinsic apoptosis in human hepatocarcinoma (HepG2) cells [54].

## 6. Effect of Neem as Anti-Inflammatory

Plants or their isolated derivatives are in the practice to treat/act as anti-inflammatory agents. A study result has confirmed that extract of* A. indica* leaves at a dose of 200 mg/kg, p.o., showed significant anti-inflammatory activity in cotton pellet granuloma assay in rats [55]. Other study results revealed that neem leaf extract showed significant anti-inflammatory effect but it is less efficacious than that of dexamethasone [56] and study results suggest that nimbidin suppresses the functions of macrophages and neutrophils relevant to inflammation [57].

Earlier finding showed immunomodulator and anti-inflammatory effect of bark and leave extracts and antipyretic and anti-inflammatory activities of oil seeds [58, 59]. Experimentation was made to evaluate the analgesic activity of neem seed oil on albino rats and results of the study showed that neem seed oil showed significant analgesic effect in the dose of 1 and 2 mL/kg and oil has dose-dependent analgesic activity [60].

Another study was made to investigate the anti-inflammatory effect of neem seed oil (NSO) on albino rats using carrageenan-induced hind paw edema and results revealed that NSO showed increased inhibition of paw edema with the progressive increase in dose from 0.25 mL to 2 mL/kg body weight. At the dose of 2 mL/kg body weight, NSO showed maximum (53.14%) inhibition of edema at 4th hour of carrageenan injection [61].

Results of the study concluded that the treated animals with 100 mg kg^−1^ dose of carbon tetrachloride extract (CTCE) of* Azadirachta indica* fruit skin and isolated ingredient azadiradione showed significant antinociceptive and anti-inflammatory activities [62].

## 7. Hepatoprotective Effect

Medicinal plants and their ingredients play a pivotal role as hepatoprotective without any adverse complications. A study was performed to investigate the hepatoprotective role of azadirachtin-A in carbon tetrachloride (CCl_4_) induced hepatotoxicity in rats and histology and ultrastructure results confirmed that pretreatment with azadirachtin-A dose-dependently reduced hepatocellular necrosis [63]. Furthermore results of the study show that pretreatment with azadirachtin-A at the higher dose levels moderately restores the rat liver to normal [63].

Another study was carried out to evaluate the protective effect of active constituent of neem such as nimbolide against carbon tetrachloride (CCl_4_) induced liver toxicity in rats and results suggest that nimbolide possesses hepatoprotective effect against CCl_4_ induced liver damage with efficiency similar to that of silymarin standard [64] and another study finding revealed that leaf extract was found to have protection against paracetamol-induced liver necrosis in rats [65].

A study assesses the hepatoprotective activity of* Azadirachta indica* (AI) leaf extract on antitubercular drugs-induced hepatotoxicity and results confirmed aqueous leaf extract significantly prevented changes in the serum levels of bilirubin, protein, alanine aminotransferase, aspartate aminotransferase, and alkaline phosphatase and significantly prevented the histological changes as compared to the group receiving antitubercular drugs [66]. Additionally, other results showed that ethanolic and aqueous leaf extracts of* A. indica* exhibited moderate activity over carbon tetrachloride treated animals [67]. Hepatoprotective effect of methanolic and aqueous extracts of* Azadirachta indica* leaves was evaluated in rats and study result established that the plant has good potential to act as hepatoprotective agent [68].

An experiment was made to investigate the protective effect of neem extract on ethanol-induced gastric mucosal lesions in rats and results showed that pretreatment with neem extract showed protection against ethanol-induced gastric mucosal damage [69].

## 8. Wound Healing Effect

Numerous plants/their constituents play an important role in the wound healing effect. A study was made to evaluate the wound healing activity of the extracts of leaves of* A. indica* and* T. cordifolia* using excision and incision wound models in Sprague Dawley rats and results revealed that extract of both plants significantly promoted the wound healing activity in both excision and incision wound models [70]. Furthermore, in incision wound, tensile strength of the healing tissue of both plants treated groups was found to be significantly higher as compared to the control group [69]. Other results showed that leave extracts of* Azadirachta indica* promote wound healing activity through increased inflammatory response and neovascularization [71].

## 9. Antidiabetic Activity

A study was undertaken to evaluate the 70% alcoholic neem root bark extract (NRE) in diabetes and results showed that neem root bark extract showed statistically significant results in 800 mg/kg dose [72]. Another experiment was performed to examine the pharmacological hypoglycemic action of* Azadirachta indica* in diabetic rats and results showed that in a glucose tolerance test with neem extract 250 mg/kg demonstrated glucose levels were significantly less as compared to the control group and* Azadirachta indica* significantly reduce glucose levels at 15th day in diabetic rats [73].

Studies using* in vivo* diabetic murine model,* A. indica*, and* B. spectabilis* chloroform, methanolic, and aqueous extracts were investigated and results showed that* A. indica* chloroform extract and* B. spectabilis* aqueous, methanolic extracts showed a good oral glucose tolerance and significantly reduced the intestinal glucosidase activity [74]. Another important study suggested that leaves extracts of* Azadirachta indica* and* Andrographis paniculata* have significant antidiabetic activity and could be a potential source for treatment of diabetes mellitus [75].

## 10. Antimicrobial Effect

Neem and its ingredients play role in the inhibition of growth of numerous microbes such as viruses, bacteria, and pathogenic fungi. The role of neem in the prevention of microbial growth is described individually as follows.

### 10.1. Antibacterial Activity

A study was performed to evaluate antimicrobial efficacy of herbal alternatives as endodontic irrigants and compared with the standard irrigant sodium hypochlorite and finding confirmed that leaf extracts and grape seed extracts showed zones of inhibition suggesting that they had antimicrobial properties [76]. Furthermore, leaf extracts showed significantly greater zones of inhibition than 3% sodium hypochlorite [76].

The antibacterial activity of guava and neem extracts against 21 strains of foodborne pathogens was evaluated and result of the study suggested that guava and neem extracts possess compounds containing antibacterial properties that can potentially be useful to control foodborne pathogens and spoilage organisms [77].

Another experiment was made to evaluate the antibacterial activity of the bark, leaf, seed, and fruit extracts of* Azadirachta indica* (neem) on bacteria isolated from adult mouth and results revealed that bark and leaf extracts showed antibacterial activity against all the test bacteria used [78]. Furthermore, seed and fruit extracts showed antibacterial activity only at higher concentrations [78].

### 10.2. Antiviral Activity

Results showed that neem bark (NBE) extract significantly blocked HSV-1 entry into cells at concentrations ranging from 50 to 100 *μ*g/mL [78]. Furthermore, blocking activity of NBE was noticed when the extract was preincubated with the virus but not with the target cells suggesting a direct anti-HSV-1 property of the neem bark [79].

Leaves extract of neem (*Azadirachta indica* A. Juss.) (NCL-11) has shown virucidal activity against coxsackievirus virus B-4 as suggested via virus inactivation and yield reduction assay besides interfering at an early event of its replication cycle [80].

### 10.3. Antifungal Activity

Experiment was made to evaluate the efficacy of various extracts of neem leaf on seed borne fungi* Aspergillus* and* Rhizopus* and results confirmed that growth of both the fungal species was significantly inhibited and controlled with both alcoholic and water extract. Furthermore, alcoholic extract of neem leaf was most effective as compared to aqueous extract for retarding the growth of both fungal species [81]. Another finding showed the antimicrobial role of aqueous extracts of neem cake in the inhibition of spore germination against three sporulating fungi such as* C. lunata*,* H. pennisetti*, and* C. gloeosporioides* f. sp.* mangiferae* [82] and results of the study revealed that methanol and ethanol extract of* Azadirachta indica* showed growth inhibition against* Aspergillus flavus*,* Alternaria solani*, and* Cladosporium* [83].

Aqueous extracts of various parts of neem such as neem oil and its chief principles have antifungal activities and have been reported by earlier investigators [84–86]. A study was undertaken to examine the antifungal activity of* Azadirachta indica* L. against* Alternaria solani* Sorauer and results confirmed that ethyl acetate fraction was found most effective in retarding fungal growth with MIC of 0.19 mg and this fraction was also effective than fungicide (metalaxyl + mancozeb) as the fungicide has MIC of 0.78 mg [87].

### 10.4. Antimalarial Activity

Experiment was made to evaluate the antimalarial activity of extracts using* Plasmodium berghei* infected albino mice and results revealed that neem leaf and stem bark extracts reduced the level of parasitemia in infected mice by about 51–80% and 56–87%, respectively, [88] and other studies showed that azadirachtin and other limonoids available in neem extracts are active on malaria vectors [89–91].

Another finding based on crude acetone/water (50/50) extract of leaves (IRAB) was performed to evaluate the activity against the asexual and the sexual forms of the malaria parasite,* Plasmodium falciparum*,* in vitro* and results showed that, in separate 72-hour cultures of both asexual parasites and mature gametocytes treated with IRAB (0.5 microg/mL), parasite numbers were less than 50% of the numbers in control cultures, which had 8.0% and 8.5% parasitemia, respectively [92].

## 11. Role of Neem in Dentistry

A study was made to assess the efficacy of neem based on mouth rinse regarding its antigingivitis effect and study confirmed that* A. indica* mouth rinse is equally effective in reducing periodontal indices as chlorhexidine [93].

Another study was carried out to evaluate the antimicrobial properties of organic extracts of neem against three bacterial strains causing dental caries and results showed that petroleum ether and chloroform extract showed strong antimicrobial activity against* S. mutans*. Chloroform extract showed strong activity against* Streptococcus salivarius* and third strain* Fusobacterium nucleatum* was highly sensitive to both ethanol and water extract [94]. Earlier finding confirmed that dried chewing sticks of* neem* showed maximum antibacterial activity against* S. mutans* as compared to* S. salivarius*,* S. mitis*, and* S. sanguis* [95].

## 12. Antinephrotoxicity Effect

An experiment was made to investigate the effects of methanolic leaves extract of* Azadirachta indica* (MLEN) on cisplatin- (CP-) induced nephrotoxicity and oxidative stress in rats and results confirmed that extract effectively rescues the kidney from CP-mediated oxidative damage [90]. Furthermore, PCR results for caspase-3 and caspase-9 and Bax genes showed downregulation in MLEN treated groups [96].

## 13. Neuroprotective Effects

A study was performed to investigate the neuroprotective effects of* Azadirachta indica* leaves against cisplatin- (CP-) induced neurotoxicity and results showed that morphological findings of neem before and after CP injection implied a well-preserved brain tissue. No changes, in biochemical parameters, were observed with neem treated groups [97].

## 14. Immunomodulatory and Growth Promoting Effect

Experiment was performed to investigate growth promoting and immunomodulatory effects of neem leaves infusion on broiler chicks and results showed that neem infusion successfully improved antibody titre, growth performance, and gross return at the level of 50 mL/liter of fresh drinking water [98].

Another study investigated the effects of feeding of powdered dry leaves of* A. indica* (AI) on humoral and cell mediated immune responses, in broilers and results showed that AI (2 g/kg) treatment significantly enhanced the antibody titres against new castle disease virus (NCDV) antigen [99].

## 15. Safety, Toxicities, and LD_50_ Values of Neem

The measurement of toxicities of natural compound is crucial before their application in health management. Various studies based on animal model and clinical trials confirmed the neem is safe at certain dose and on the other side neem and its ingredients showed toxic/adverse effect.

Several studies reported, in children, neem oil poisoning causing vomiting, hepatic toxicity, metabolic acidosis, and encephalopathy [100–102] and another study based on rat model showed that administration of leaf sap caused an antianxiety effect at low doses, whereas high doses did not show such types of effect [103]. An important study based on rats model showed that azadirachtin did not show toxicity even at 5 g/kg bw [104]. A study based on rabbit was performed to check the toxicological analysis and results of the study showed there was progressive increase in body weight in both the test and control animals, and during the entire duration of the administration of the neem extract, there was no observed sign of toxicity in both groups [105].

A study result showed that, in the acute toxicity test, the LD_50_ values of neem oil were found to be 31.95 g/kg [106]. Another study was performed to evaluate the toxicity in chicken and finding showed that acute toxicity study of neem leaf aqueous extract revealed an intraperitoneal LD_50_ of 4800 mg/kg, and clinical signs were dose dependent [107].

A study reported that lethal median doses (LD_50_) recorded for neem leaf and stem bark extracts were 31.62 and 489.90 mg/kg body weight, respectively [108]. The LD_50_ of water extract of* A. indica* leaves and seeds were 6.2, 9.4 mL kg^−1^, respectively [109]. Lethal dose values were calculated with probit analysis and LD_50_ and LD_90_ values were found to be 8.4 and 169.8 *µ*g/fly of neem extract, respectively [110]. A test for acute oral toxicity in mice revealed that LD_50_ value of approximately 13 g/kg body weight [111].

## 16. Clinical Studies Based on Neem

Various clinical trials based studies confirmed that herbal products or derivatives from the natural products play vital role in diseases prevention and treatment. A very few studies on active compounds such as nimbidin were made to check the efficacy in the health management. An important study was made based on human subjects to investigate the role of neem bark extract as antisecretory and antiulcer effects in human subjects. Administration of lyophilised powder of the extract for 10 days at the dose of 30 mg twice daily showed significant decrease (77%) of gastric acid secretion. The bark extract at the dose of 30–60 mg twice daily for 10 weeks almost completely healed the duodenal ulcers and one case of esophageal ulcer and one case of gastric ulcer healed completely when administrated at the dose of 30 mg twice daily for 6 weeks [9].

A double blind clinical drug trial study was performed to check the efficacy of drug made up of aqueous extract of neem leaves in 50 cases of uncomplicated psoriasis taking conventional coal tar regime and results revealed that patients taking drug in addition to coal tar had shown a quicker and better response in comparison to placebo group [112]. A clinical study of six weeks was made to check the efficacy of neem extract dental gel with chlorhexidine gluconate (0.2% w/v) mouthwash as positive control and results of the study showed that the dental gel containing neem extract has significantly reduced the plaque index and bacterial count compared to that of the control group [113]. A study showed that, in ulcer healing tests, nimbidin significantly enhanced the healing process in acetic acid induced chronic gastric lesions in albino rats and dogs [114].

## 17. Conclusion

Popularity of natural products or their derivatives role in diseases cure and prevention is increasing worldwide due to less side effect properties. Neem and its ingredients have therapeutics implication and have been traditionally used worldwide especially in Indian Subcontinent since ancient time. Clinical based studies confirmed that neem plays pivotal role in prevention of various diseases. The role of active ingredients as chemopreventive effect has been noticed in various tumour via modulation of numerous cell signaling pathways. The detailed study should be made based on animal to know the exact mechanism of action in the diseases management.

## Figures and Tables

**Table 1:** Taxonomic position of *Azadirachtaindica* (neem).

Order	Rutales

Suborder	Rutinae

Family	Meliaceae

Subfamily	Melioideae

Tribe	Melieae

Genus	*Azadirachta *

Species	*indica*
